# Impact of transcranial alternating current stimulation on psychological stress: A functional near-infrared spectroscopy study

**DOI:** 10.1371/journal.pone.0319702

**Published:** 2025-03-26

**Authors:** M. N. Afzal Khan, Yara Badr, Sandra Mary Prasad, Usman Tariq, Fadwa Almughairbi, Fabio Babiloni, Fares Al-Shargie, Hasan Al-Nashash

**Affiliations:** 1 Department of Electrical Engineering, American University of Sharjah, Sharjah, United Arab Emirates; 2 Biosciences and Bioengineering Graduate Program, American University of Sharjah, Sharjah, United Arab Emirates; 3 Department of Cognitive Sciences, United Arab Emirates University, Abu Dhabi, United Arab Emirates; 4 Department Molecular Medicine, University of Sapienza Rome, Rome, Italy; 5 Rutgers University, Newark, United State of America; Air University, PAKISTAN

## Abstract

This pilot study investigates the impact of transcranial alternating current stimulation (tACS) on psychological stress using functional near-infrared spectroscopy (fNIRS). Forty volunteers were randomly assigned to two groups: the tACS and the control. The experiment was divided into three distinct stages: pre-stimulation, stimulation, and post-stimulation. The Stroop Color-Word Task (SCWT) was employed as a validated stress-inducing paradigm to assess pre- and post-stimulation changes. During the initial phase, the participants completed the SCWT. This was followed by either tACS or sham. In the third session, the individuals solved the task again. The anode and cathode for the transcranial tACS were placed on the dorsolateral prefrontal cortex (DLPFC). tACS, was applied with current intensity of 1.5 mA at 16 Hz over the dorsolateral prefrontal cortex (DLPFC), aimed to modulate cortical activation and mitigate stress. Sham included 5-second ramp periods. Physiological data using alpha amylase and the NASA Task Load Index (NASA-TLX) were utilized. The results revealed significant hemodynamic changes and reduced stress levels in the tACS group compared to the sham group (*p* <  0.001). The connectivity network changed significantly (*p* <  0.001) following tACS. In addition, the NASA-TLX results showed a statistically significant difference between the pre-and post-tACS sessions. In contrary, no statistical significance was noticed for the sham control group. An increase in the blood flow in the prefrontal cortex region of the brain was observed, demonstrating the potential of tACS as a non-invasive neuromodulation technique for stress mitigation.

## 1. Introduction

Transcranial electric stimulation (tES) encompasses a range of noninvasive methods, such as transcranial direct current stimulation (tDCS), transcranial alternating current stimulation (tACS), and transcranial random noise stimulation. Such approaches are extensively utilized in the fields of clinical and cognitive sciences [1]. These techniques involve the use of low-intensity direct current (DC) or alternating current (AC), usually between 0.5 and 2 mA, to stimulate the brain by applying it to the scalp using surface electrodes. tES is commonly used in clinical and cognitive studies to specifically stimulate the prefrontal cortex (PFC). Altering the activity in the dorsolateral prefrontal cortex (DLPFC) has demonstrated promise in improving cognitive abilities like as memory, attention, and multitasking, as well as in treating mental conditions such as major depressive disorder and autism [2–5].

The process of tDCS includes modulating neuronal activity by adjusting the resting membrane potential towards depolarization or hyperpolarization, depending on the specific type of stimulation [6]. It is claimed that anodal tDCS can increase the firing rate of neurons located under the stimulated electrode, leading to improved performance in mental tasks [7,8]. On the other hand, cathodal tDCS decreases the activity of neurons, leading to a decline in task performance [9–11]. Research has shown that electrical stimulation may alter the patterns of brain connection, impacting both the stimulated area and other areas [12,13]. For example, studies have demonstrated that tDCS can alter the functional connectivity of frontoparietal networks that are engaged in cognitive tasks by specifically targeting the DLPFC [13].

While tDCS operates by modulating the resting membrane potential to change neural excitability, tACS shows its effects by applying alternating currents at specific frequencies to entrain brain oscillations. This ability to synchronize neural activity with oscillatory electrical fields is particularly advantageous for addressing irregularities in brain function, such as those observed during mental stress. In the context of this study, tACS was selected due to its potential to target the DLPFC area and modulate stress-related oscillatory activity, which has been implicated in stress regulation and cognitive control.

Meanwhile, tACS is employed to regulate brain oscillations while engaging in cognitive tasks [14,15], with the potential to target irregularities identified in individuals with neuropsychiatric conditions [16]. This method enables the alteration of brain oscillations by injecting currents of 0.5-2 mA that are coordinated with biological rhythms through the scalp [17,18]. It can potentially treat disorders characterized by abnormal oscillatory patterns (oscillopathies) [19]. Extensive research on tACS has thoroughly investigated its impact on several cognitive abilities such as working memory, perception, multitasking, motor control, and learning [20–30]. Several studies have explicitly investigated the physiological changes that occur during the resting state after tACS [20,27].

Integrating electrical stimulation with functional imaging methods has the potential to provide useful insights into the neuromodulatory effects of tES. Researchers have documented changes in the way blood flows, the chemicals that convey signals in the brain, and the activity of the neurons because of electric fields generated by tES [31,32]. Furthermore, studies have demonstrated that tDCS and transcranial infrared laser stimulation can alter cerebral blood flow [33]. Nevertheless, the complete understanding of the effects of tACS that targets both hemispheres of the PFC on hemodynamics is yet to be achieved. The precise mechanism responsible for the aftereffects caused by tACS is a subject of discussion, primarily because various studies have detected variations in stimulation settings. The factors addressed in this context are duration, frequency, intensity, and electrode configuration, in addition to a limited comprehension of the neurophysiological mechanisms at play. Additionally, the precision of electroencephalography (EEG) readings might be impaired during tES as a result of electrical abnormalities. Furthermore, the understanding of the hemodynamic changes that occur during stimulation, specifically in relation to tACS, is still inadequate.

One potential application for using electric stimulation is mental stress mitigation. Stress, which may be characterized as a condition of psychological strain or unease, is an inherent reaction that differs among individuals and environments. Work-related stress, frequently triggered by an overwhelming amount of work and unachievable timeframes and targets, can result in notable health problems like disrupted sleep, migraines, reduced focus, and higher rates of absenteeism [34]. These stress-induced issues not only impact personal welfare but also lead to significant financial losses for enterprises as a result of decreased production and higher healthcare expenses [35].

Functional near-infrared spectroscopy (fNIRS) known for its mobility and noninvasive nature is used to measure the changes in hemoglobin concentration with relatively high spatial and temporal resolution. The increasing use of fNIRS in functional neuroimaging research is likely due to its cost-effectiveness and mobility in comparison to functional magnetic resonance imaging (fMRI). Unlike fMRI, fNIRS unobtrusively tracks fluctuations in cerebral blood flow, without requiring subjects to be in a reclined position. This technique provides superior temporal resolution and eliminates limitations related to space constraints. The aforementioned attributes make fNIRS well-suited for investigating changes in blood flow linked to cerebral function in a wide range of contexts, including real-life surroundings.

With its versatility as a neuroimaging technique, fNIRS has shown great potential and has been applied in different disciplines. Advantages of this method over electroencephalography include superior spatial resolution and less sensitivity to noise. fNIRS has been utilized in a wide range of research studies, including investigations into neurodevelopment, cognitive processing, psychiatric disorders, language research, stroke recovery, clinical imaging, Brain-Computer Interfaces, and mental stress analysis [36–40].

Multiple studies have used fNIRS as a main instrument to examine cortical activity in different brain areas under varied stress circumstances [41,42]. An example is a research conducted in 2018 that examined the influence of mental stress on occupational performance. The study found that higher activity of the prefrontal cortex is an indicator of stress [43]. In a subsequent investigation, the Trier Social Stress Test was employed to investigate the impact of psychosocial stress on cognitive function in adolescent males. This work emphasized the significance of fNIRS in evaluating stress responses [44]. Researchers have utilized mental arithmetic activities and other stress-inducing stimuli to successfully deploy fNIRS for the early identification and quantification of mental stress [45,46]. These studies highlight the effectiveness of fNIRS as a neuroimaging technique for examining and measuring mental stress in various stress scenarios. Studies have shown that PFC activation is affected when a person is under stress [47] and improvement in the PFC activation leads to mitigation of stress [48].

Several review articles have been recently published summarizing the studies on mental comparing modern deep learning methods used to evaluate mental stress [47,48]. Alongside the work done on mental stress detection, significant progress has been made in exploring techniques to mitigate stress. One of the prominent ways utilized to mitigate mental stress is through sound. A recently published study has proven the efficacy of the use of pure tone in this regard [49]. Recently binaural beats have also shown promising effects for the mitigation of mental stress [50,51]. However, to the best of the authors’ knowledge, to date the literature lacks in addressing tACS affects mental stress. Building up prior studies in the field, the present work investigates the application of tACS for mitigating mental stress, aiming to address this critical research gap.

Keeping in view the knowledge gap, in this pilot study, a comparison is made between the effects of tACS on the PFC of persons who are experiencing mental stress utilizing fNIRS as an imaging tool. Simultaneously, the study examines how the brain reacts in similar conditions for the sham group. Building on previous work, our hypothesis posited that tACS would mitigate stress. The study also discusses the impact of tACS on hemodynamics and its influence on mental stress. The results of the study show a higher activation in the PFC of tACS group as compared to sham group. This finding is also reflected in the feedback taken from participants pre- and post-stimulation. To summarize, we examined the changes in blood flow dynamics prior to and following the application of tACS and a placebo treatment (sham). The analysis primarily relies on comparing hemodynamic changes, brain activation patterns, and connectivity analysis.

## 2. Materials and methods.

### 2.1. Participants

A total of 40 individuals, consisting of both male (*n* =  25) and female (*n* =  15) participants, who were either employed or enrolled as students at the American University of Sharjah, took part in this research. The participants’ visual acuity ranged from normal to corrected to normal. All subjects indicated no difficulties in perceiving auditory or visual stimuli pertaining to color. None of the subjects had a previous medical history of neurological or visual impairment, and there was no evidence of drug addiction or ongoing medication usage. On the day of the experiment, the volunteers were given instructions to abstain from consuming any alcohol, caffeine, or other beverages that would enhance their energy levels. Before commencing the experiment, each participant received a thorough explanation on the study and was provided with the option to terminate their participation at any given point. A consent form was signed before the experiment commenced. The study was conducted in accordance with the latest Helsinki Declaration, after obtaining authorization from the Institutional Review Board (IRB) of the American University of Sharjah [52].

### 2.2. Stressor design

This study employed the Stroop Color-Word Task (SCWT) as a method to induce stress. SCWT is one of the well-known and widely used stressor in literature [53,54]. As part of the task, participants were required to focus on twelve specific color words that came up in a random sequence: “Orange,” “Purple,” “Mustard,” “Indigo,” “Dark Green,” “Maroon,” “Green,” “Yellow,” “Red,” “Cyan,” “Magenta,” and “Blue.”

The word displayed on the computer screen was printed in a color that did not match its literal meaning. The participants were presented with a cognitive test where they had to choose the color of the written word rather than the word itself. Participants were required to match the ink color from a set of twelve options shown as push buttons underneath the displayed word. The buttons had a backdrop color that was distinct from the other colors, and the text on the buttons was shown in a separate color to make the task more challenging. Instead of determining the color of the button itself, participants were instructed to identify the color written inside it.

**Fig 1:**
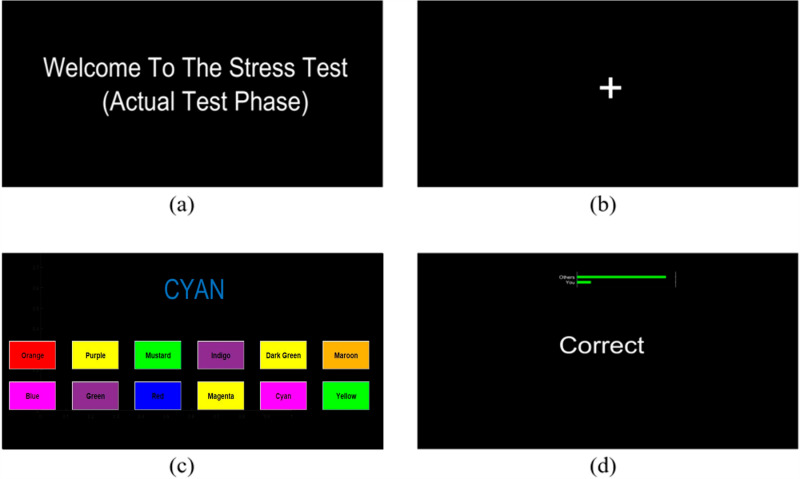
Illustration of the Stroop Color-Word Task utilized as a stressor in this study: (a) Introduction screen (b) Relaxation phase (c) SCWT task (d) Confirmation message for correct selection.

To ensure increasing task difficulty, the response time limited at 3 seconds. Each question was subject to this time constraint, and if a participant failed to provide a response within the allocated timeframe, a message stating “Time is out” would be shown on the screen. Participants received feedback on the accuracy of their chosen option shown on the screen. The SCWT protocol was implemented using MATLAB®, ensuring precision and uniformity in task performance. Each SCWT session consisted of 16 trials. The task period for each trial consisted of 7 questions, with each question lasting approximately 3 seconds based on the time constraints. Participants were given a practice trial prior to the experiment to familiarize themselves with the protocol. To validate the stress-inducing nature of the SCWT, participants were asked to fill out questionnaire. Fig 1 depicts a sample question that participants viewed on their screen.

### 2.3. Experimental paradigm and electrical stimulation

**Fig 2:**
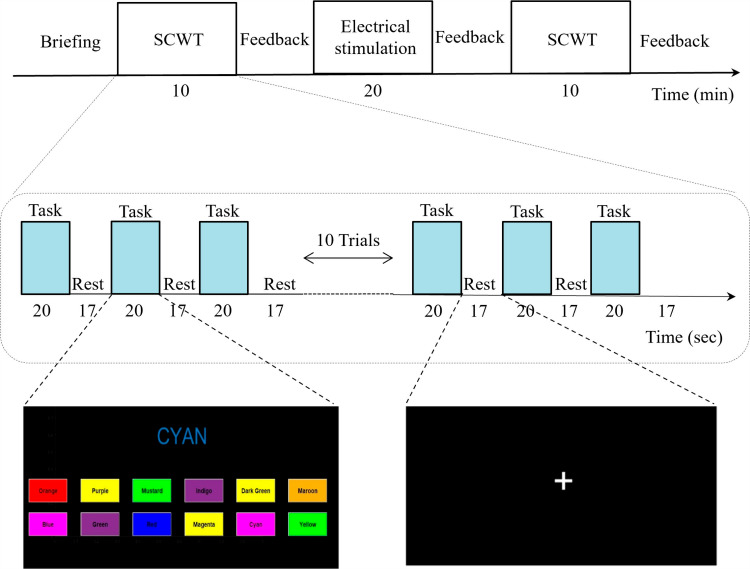
Experimental paradigm for fNIRS recording. SCWT, Stroop Color Word Task.

**Fig 3:**
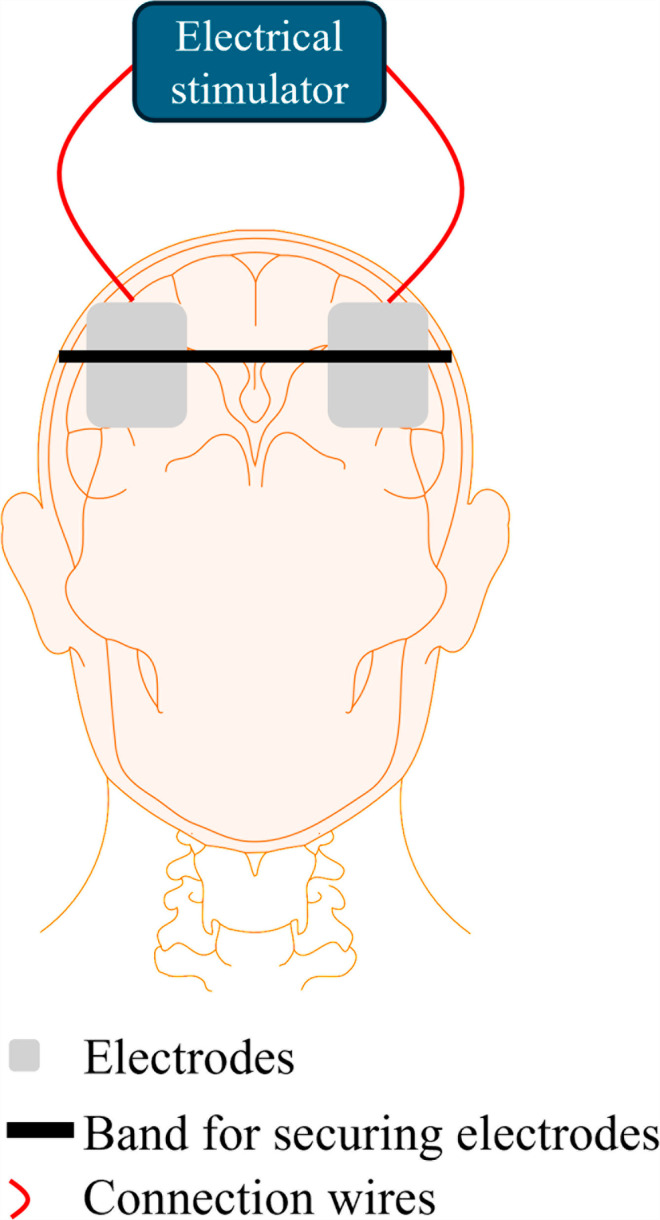
tES Electrode configuration.

The study took place in three sessions, namely, pre-stimulation, stimulation, and post-electrical stimulation. First and third sessions of the experiment consisted of sixteen trials, with each trial lasting thirty-seven seconds, including a task time of twenty seconds. In between the two sessions, the subjects received tACS stimulation for a duration of twenty minutes. Fig 2 provides a concise overview of the experimental paradigm.

battery-powered Starstim tES system (Neurostim, Neurosoft) was used to deliver electrical stimulation. The tACS system consists of two electrodes, one serving as an anode and the other as a cathode. The electrode configuration was positioned over the DLPFC region of the head. Both electrodes were composed of conductive silicon and had a surface area of 2.5 cm2. The electrodes were affixed to the skin using a gel-filled foam that enhances conductivity. The electrodes were positioned on the PFC with the anode located approximately 6 cm apart from the returning electrode, as shown in Fig 3. The current capacity of tACS provided was 1.5 mA amplitude (i.e., the zero-to-peak) at 16 Hz. The stimulation parameters were chosen in line with the published literature [5,23]. The tES system was wired to a computer. The study utilized a software program to create the stimulation routine, which involved placing one anode and one cathode.

Participants were seated in a comfortable chair and given instructions to minimize body movement during the experiment. In both sessions of the experiment, a consistent inter-stimulation interval of 17 seconds was maintained, along with pre-and post-rest periods of 20 seconds each. During the rest period, a black screen was displayed. The participants were presented with visual stimuli on a computer screen and were asked to keep their eyes open for the duration of the experiment. For the time during stimulation, the participants were asked to relax but not to sleep.

### 2.4. Optode placement

**Fig 4:**
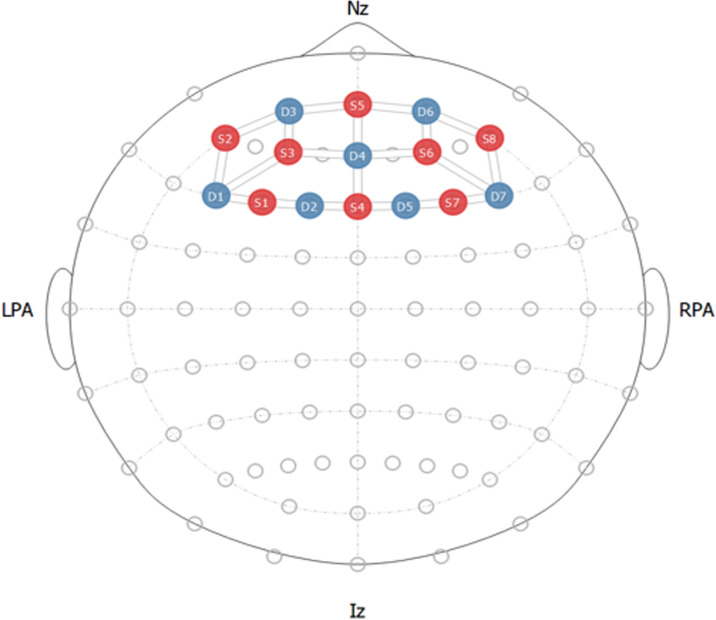
fNIRS optode configuration with red dots showing sources and blue showing detectors.

Seven detectors and eight emitters were utilized to record brain activity in the prefrontal cortex region. Fig 4 displays the optode setup on the region of interest. The FpZ area of the brain was selected as the reference point to ensure precise positioning on the prefrontal cortex. The selection of this reference point was based on the International 10-20 System, which ensures accurate placement of the electrodes.

### 2.5. fNIRS Data acquisition and pre-processing

The study involved sampling brain signals (fNIRS data) at a frequency of 10.17 Hz. The fNIRS signals were acquired using a single-phase continuous wave fNIRS device, NIRSport2 system from NIRx Medical Technologies. The system utilized two distinct wavelengths: 760 nm and 850 nm. The conversion of raw intensities into changes in ΔHbO and ΔHbR was accomplished by employing the Modified Beer-Lambert Law [55]. The data from the light intensity was converted to changes in hemoglobin using NIRSlab. Investigators have utilized various techniques to preprocess the fNIRS data [56]. Following the acquisition, the data (ΔHbO and ΔHbR) for the current study underwent preprocessing to eliminate any noise contamination that may have affected the quality of the signal. In order to address any distortions in the signals caused by the mobility of the subjects, the converted intensities of ΔHbO and ΔHbR were initially examined employing principal component analysis and temporal derivative distribution correction [57,58]. After applying motion-artifact correction, a Butterworth filter was used to eliminate cardiac, respiratory, and low-frequency drift signals. The filter had a low-pass cutoff frequency of 0.15 Hz and a high-pass cutoff frequency of 0.01 Hz. Finally, this study was fitted to the desired hemodynamic response function (dHRF) to detect neural activity. The dHRF was generated by applying two gamma functions [59].

### 2.6. Physiological measurement

Studies addressing stress often fail to consider the biological factors that might be influencing the human response. This leads to inability to fully explain the variations in human performance under stress [60]. It’s important to evaluate human performance by considering both task outcomes and the physiological effort required to achieve them [61]. Physiological indicators, which can reflect cognitive effort, include pupil dilation [62], heart rate [63], and saliva [64].

In this paper, to measure the stress levels a total of four saliva samples were collected in each experiment (i.e., for both tACS group and for sham group). First sample was collected before the start of the experiment, second sample was collected after the first session of SCWT ended and before the electrical stimulation session started, third sample was collected at the end of the electrical stimulation session, and fourth sample was collected at the end of experiment (i.e., after the second SCWT session).

The saliva samples were collected using COCORO meter (Nipro Co., Osaka, Japan). For each of the four samples, the participants had to insert a new strip in their mouth for approximately 40 seconds. Then this strip was placed in the COCORO meter to get the stress level measurement. The COCORO meter showed the value of stress after analyzing the enzyme levels in saliva.

### 2.7. Subjective measurement using NASA task load index

Developed by NASA Ames Research Center in the 1980s, the NASA Task Load Index (NASA TLX) system is used to subjectively evaluate the workload or stress of human operators interacting with human-machine systems [65–68]. The NASA TLX consists of a self-report questionnaire which measures overall workload. The self-report questionnaire contains six questions, rated on a scale from 1 to 20, which assess perceived levels of workload and stress. These questions cover six subscales: mental demand, physical demand, temporal demand, frustration, effort, and performance. In the NASA TLX form, participants rate performance from “perfect” to “failure” and other aspects from “very low” to “very high” [69].

In this study, the participants were asked to fill-up the NASA TLX forms twice in each experiment. The first form was filled in by participants at the end of the first session of SCWT. The second form was filled in at the end of the experiment (i.e., after the SCWT session post electrical stimulation).

### 2.8. Statistical analysis

After pre-processing, the data was analysed and compared in three ways, i.e., using averaged time series, brain activation maps, and through connectivity analysis. For the comparison of time series data, a statistical method was used. The mean of ΔHbO was computed by using *t*-values, and *p*-values for statistical analysis and the identification of active channels. The trial period’s degree of freedom was utilized to select the *t*_crt_ value, while a significance threshold of 0.05 was kept for the one-tailed *t*-test. The *t*-values were calculated using MATLAB^®^’s *robustfit* function. An active channel was defined as having a *t*-value higher than *t*_crt_ and a *p*-value lower than 0.05. In order to detect cortical activation, the time-series signal for each trial was matched to the required hemodynamic response (dHRF) signal [70].

To conduct image-based analysis, the data from all trials were initially transformed into visual representations. Averaged activation maps and connectivity maps were generated for that purpose. *t*-test was conducted to generate activation maps, often known as *t*-maps. The *t*-values for all the channels were computed using the *robustfit* function in MATLAB. The statistical significance level of the test was again set at 0.05. In order to produce connectivity matrices, the Pearson correlation coefficients were computed for the data from each channel [71]. The connectivity matrices were subsequently utilized to generate connectivity maps.

## 3. Results

### 3.1. Safety and Feasibility of tACS

No significant deleterious effects were seen in either of the stimulation situations (sham, tACS). When comparing conditions of stimulation with each other, there were no noted differences in adverse effects, except for weariness and tingling. Statistically, the occurrence of tingling was more pronounced in the tACS group compared to the sham group (*p* <  0.05). Furthermore, based upon the feedback, the level of weariness experienced by participants in the sham group was significantly higher compared to the tACS group. In addition, nearly all of the individuals in the tACS group reported experiencing moderate to severe phosphene and bumping symptoms. This effect may be attributed to the close proximity of the stimulation/return electrodes in the area near the eyes. However, the participants in the control group did not report any phosphene impact. The participants of tACS group reported higher level of concentration after receiving the stimulation as compared to before that. On the other hand, the control group reported feeling of tiredness and boredom when they performed experiment after the sham stimulation.

### 3.2. Comparison of physiological response

**Fig 5:**
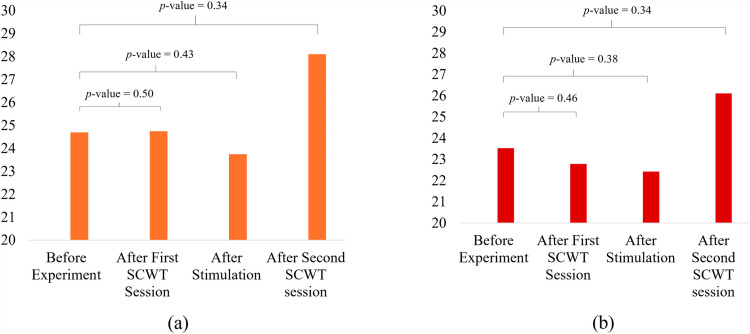
Stress levels measured using saliva samples: a) tACS group. b) Sham group. *p*-values achieved for comparison of each phase is written on top. Overall, no statistical difference was noticed in any of the comparisons.

The values achieved using saliva samples was noted for each of the participant. These values were then averaged to see the overall trend in the stress level throughout the experiment. Fig 5 shows the averaged values of the stress levels measured for tACS group and the control group. By visual inspection, for the tACS group, the average value remained similar before (24.5) and after (24.6) the first SCWT session. But the value decreased after the tACS stimulation was given (23.9). In contrary, the value drastically increased after the second session of SCWT (28.1). Almost similar trend was also noticed for the Sham group. For the sham group, before the beginning of experiment the average value came out to be 23.6 whereas after SCWT session it became 23. Following sham stimulation, the value slightly decreased to 22.8 followed by a drastic increase reaching 25.9. Moreover, no statistical significance was found in any of the readings. The *p*-values achieved for the statistical tests performed on the data are shown on top of each measurement in Fig 5.

### 3.3. Comparison of NASA TLX score

**Fig 6:**
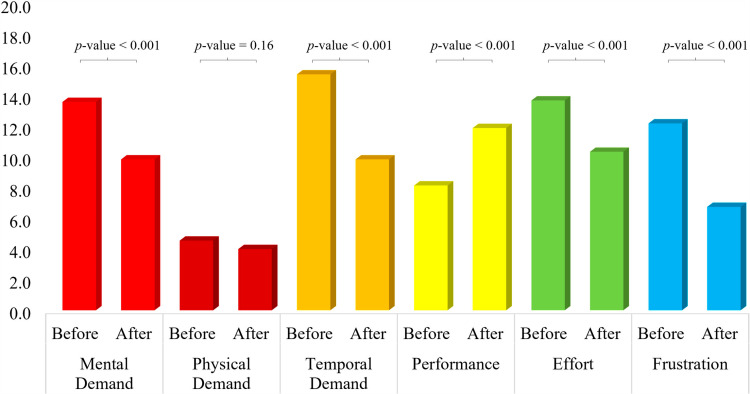
Comparison of average NASA TLX response for tACS group. Before: Responses given by participants after first session of SCWT (before tACS session). After: Responses given by participants after second session of SCWT (after tACS session). p-values achieved for comparison of before and after tACS session are written on top.

The feedback received from the participants in the NASA TLX forms was noted and analysed. Fig 6 shows the averaged value of NASA TLX responses given by the participants in tACS group. Statistical tests were performed in order to evaluate the feedback received from the participants. For NASA TLX score, a response was considered statistically significant if the *p*-value <  0.01. While comparing the mental demand, a statistically significant response was reported by participants when they performed SCWT after the tACS session. However, no statistical significance was noted while comparing the response for the physical demand. This response is in-line with our work as no physical exertion is included in the experiment. The values of temporal demand, effort, and frustration reported by participants significantly decreased when SCWT was performed after the tACS session. Moreover, statistically significant increase in the performance was also noticed by the participants after the tACS experiment. The p-values for each comparison are mentioned in Fig 6. Overall, the results are positive and in-line with the hypothesis of the work.

**Fig 7:**
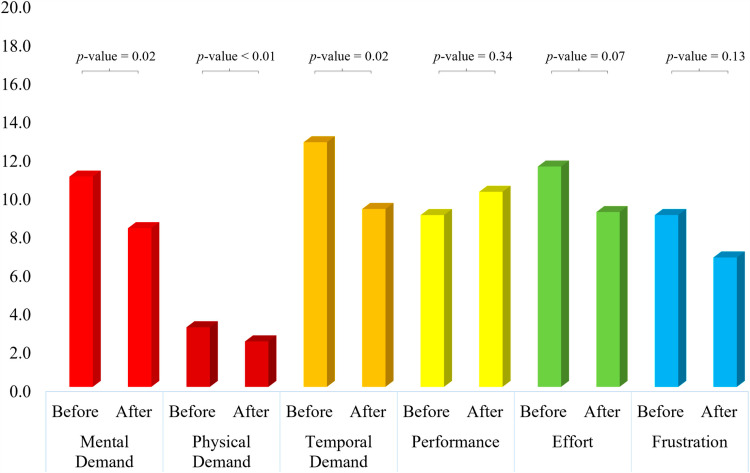
Comparison of average NASA TLX response for sham group. Before: Responses given by participants after first session of SCWT (before sham session). After: Responses given by participants after second session of SCWT (after sham session). *p*-values achieved for comparison of before and after tACS session are written on top.

Five out of six entities showed a positive response with a statistically significant improvement in response. Although, in Physical demand the average value decreased after the tACS session, but no statistically significant results were observed. Fig 7 shows the averaged value of NASA TLX responses given by the participants in the control group. While visual inspection of the figure shows a trend somewhat similar to that of tACS group in Fig 6, no statistical significance was noted in any of the six parameters except the physical demand, answered by the participants. This comparison shows the effectiveness of tACS reported by the participants themselves. None of the reposes except physical demand showed a statistically significant.

### 3.4. Comparison of hemodynamic responses

**Fig 8:**
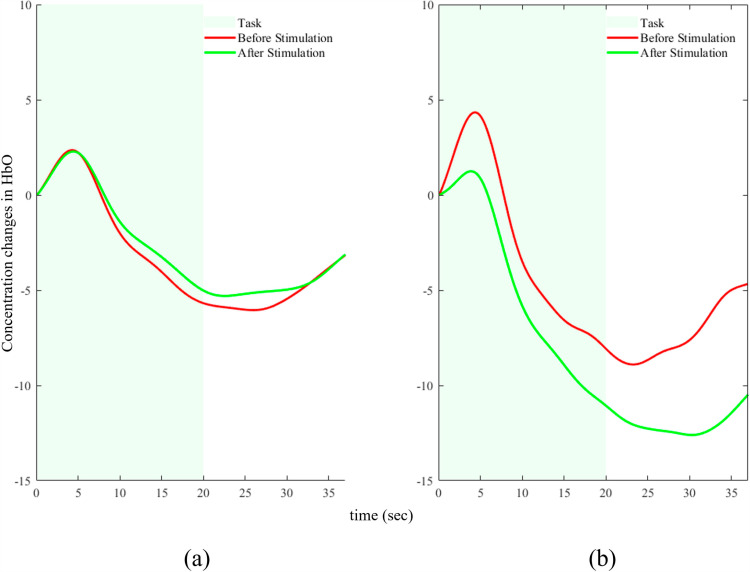
Averaged hemodynamic response for: a) tACS group. b) Sham group.

The hemodynamic response function (HRF) is an inherited pattern that is observed in the human brain for almost any kind of stimuli. The desired hemodynamic response function (dHRF) was used to identify the trials that showed activation and determine the contour of the HRF. After the extraction process, the active trials were averaged for each individual. The pre-stimulation and post-stimulation phases for both tACS and control groups followed an identical protocol of processing. Credible activation was confirmed through visual inspection in all cases. There was an increase in the hemodynamic response observed after the tACS session in comparison to the one prior to it. However, the peak value of the response was the same. Nevertheless, the brain activation following the sham session diminished significantly (*p* <  0.05). Fig 8 displays the averaged patterns of hemodynamic response for each of the two scenarios for both groups. The duration of the task is represented by the green shaded region, while the non-shaded area represents the duration of the rest.

For the tACS group the peak value achieved is the same for before and after stimulation. However, the hemodynamic response went down quickly and to a lower value before stimulation while after tACS the response was relatively improved. On the other hand, for the sham group, the peak value after sham session didn’t rise as high as before sham. Alongside, the overall hemodynamic response after sham stimulation was much lower than before stimulation. *t*-tests were employed to assess the statistical significance of the activation level for both groups. Paired t-tests were employed to examine the level of activation between pre-stimulation and post-stimulation.

A difference was observed in the average activation levels between the pre-and post-stimulation phases for both the tACS and sham stimulation methods. Notably, there was no statistically significant difference in the responses of the pre-stimulation phases for both groups (*p*-value =  0.351). Nevertheless, a notable difference was seen when comparing the post-stimulation phases of both groups (*p*-value <  0.005). In general, there was a substantial enhancement in the hemodynamic response as a result of tACS.

### 3.5. Comparison of activation patterns

**Fig 9:**
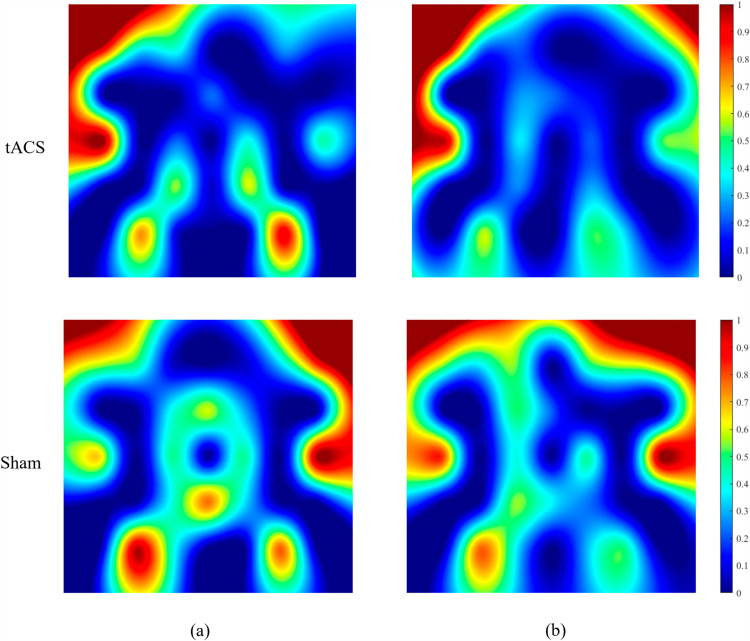
Averaged activation maps for all subjects. First row shows tACS response and second row shows sham response for: a) Pre-stimulation. b) Post-stimulation.

Fig 9 displays t-maps generated from the calculated t-values. The bar on the right side displays the range of normalized signal intensity, ranging from 0 to 1. It has been noted that tACS had an influence on the activation of the DLPFC area of the brain. Whereas, decrease in the brain activity is noticed post sham-stimulation. Fig 9 shows that the active spots/region in DLPFC region of participants became noticeably wider post-tACS as compared to the active spots/region before any external intervention. Ultimately, it is evident that the activity on the channels that were directly influenced by the electrical stimulation had higher t-values which in-turns depict better brain activation. In the tACS group the DLPFC area got more activated after the stimulation. Whereas, for the Sham group, the amount of activation stayed almost consistent.

### 3.6. Comparison of functional connectivity

**Fig 10:**
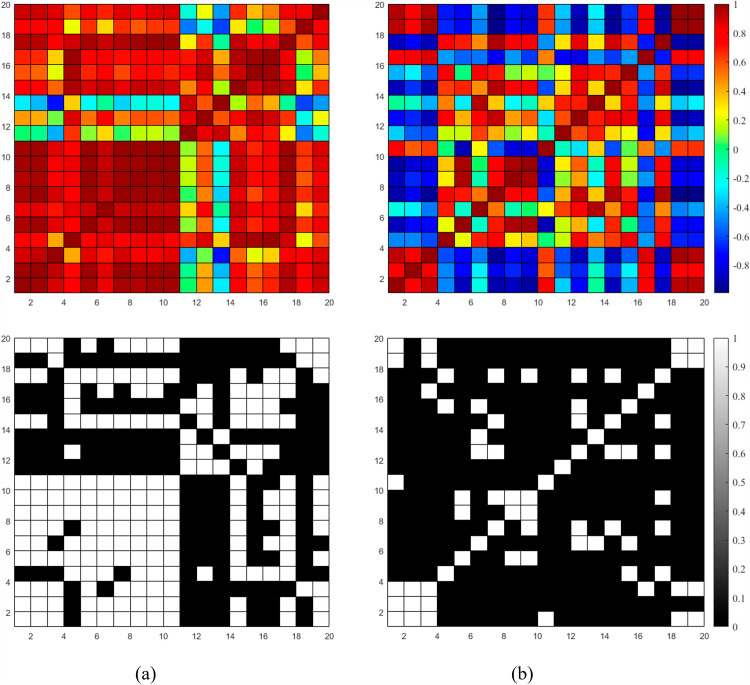
Connectivity maps and binary connectivity maps with a threshold of 0.8 for tACS group: a) Pre-stimulation. b) Post-stimulation.

**Fig 11:**
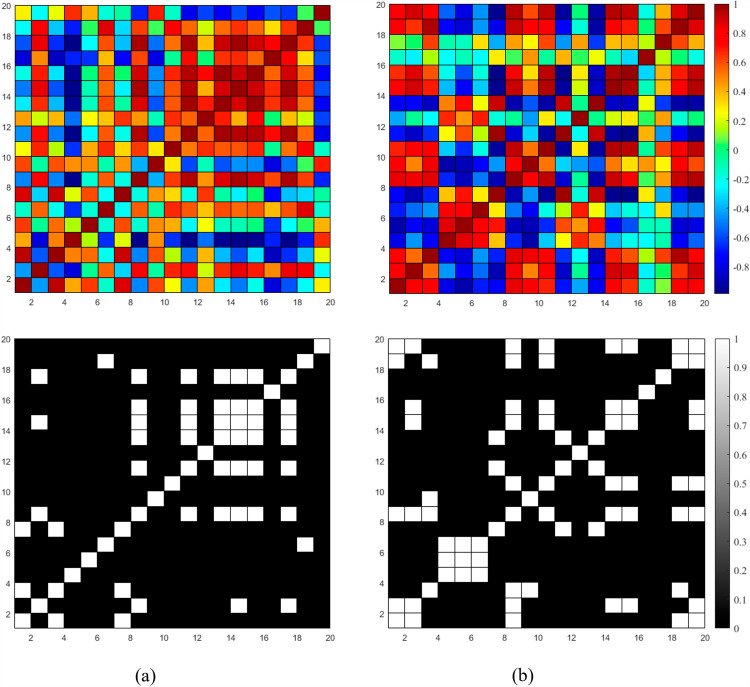
Connectivity maps and binary connectivity maps with a threshold of 0.8 for sham group: a) Pre-stimulation. b) Post-stimulation.

Figs 10 and 11 display the functional connectivity matrices and the corresponding binary connectivity matrices (with a threshold value of 0.8) for both the tACS and control groups respectively. Both figures demonstrate that there is a presence of connectivity in the situations when there is no external intervention involved. Interestingly, there was a lower level of connection in the group that received tACS compared to pre-stimulation (*p* =  0.0014, two-sample independent *t*-test) as shown in Fig 10. In contrast, the connectivity remains almost similar for the case of sham group.

## 4. Discussions

The purpose of this pilot study was to present the effects of tACS on individuals who were experiencing mental stress and to compare the results obtained with a sham control group that was condition matched. This study is the first to assess the therapeutic effects of tACS on individuals experiencing stress, utilizing fNIRS as an imaging method. SCWT is utilized as a stressor along with time. This type of stressors may offer a deeper understanding of the neurological mechanisms underlying mental stress, which has long been a central focus of neuroscience research exploring both normal brains functioning and pathological disorders.

Multiple studies have demonstrated that the flow of blood in the brain, known as hemodynamics, is interconnected in a dynamic network structure. Therefore, the utilization of functional connectivity analysis offers evidence of the impacts of tACS on the brain state of individuals experiencing stress. This explains the intricate dynamics and interactions taking place in the brain. The functional connectivity analysis revealed the alteration of connections among several channels. Furthermore, the statistical tests demonstrated substantial differences in DLPFC region post-tACS session compared to the pre-stimulation phase, with an overall upward trend observed in case of brain activation maps. Our hypothesis suggested that following a tACS session, people experiencing mental stress would exhibit more prefrontal activation during the performance of SCWT task. We anticipated a rise in the hemodynamic response in comparison to the baseline (pre-stimulation). The findings of the present study show a notable increase in hemodynamic response post-stimulation. Pre- stimulation, the hemodynamic response reached a peak value and decreased with a relatively higher slope. This demonstrates that the participant lost concentration quickly. Whereas, after the tACS session the decreasing sloop was relatively less after the peak value demonstrating that participants’ mind was more active after the stimulation session. Moreover, the maximum amount of activation was not altered due to the tACS session. The prefrontal activation was influenced by the tACS session and demonstrated a significant increase in DLPFC area activation after tACS stimulation. On the other hand, the peak value of activation significantly decreased in the sham group after the sham session. Alongside, the brain activation maps also showed no significant difference before and after sham stimulation. The decrease in response observed in the sham group (relative to pre-stimulation) can be attributed to the subjects’ tiredness over time.

The functional connection of the frontal lobe has a significant role in common mechanisms such as arithmetic, spatial thinking, and working memory activities. It is responsive to cognitive training and stress [72]. The level of connection may decrease as a result of psychological stress experienced in the workplace. Thus, we conducted a comprehensive assessment of the therapeutic impact of tACS on individuals who were subjected to generated mental stress. The findings of the functional connectivity analysis in this study offer evidence of decrease of connectivity following the tACS session. The findings of our study are corroborated by prior research, which demonstrated reduction in functional connectivity of PFC following electrical stimulation [73]. Following external intervention using tACS, a decreased in task-based functional connectivity was observed, when compared with the pre-stimulation state. In contrast, the connectivity in cases of pre and post sham stimulation remained nearly unchanged. The findings demonstrated that tACS effected the functional connectivity in the atypical frontal region associated with mental stress. The modification in functional connectivity is believed to be caused by the rebalancing effects of electrical stimulation. Overall, the results of the study demonstrate the therapeutic benefits of tACS and support its reported clinical effectiveness in literature. This imply that electrical stimulation helps maintain a homeostatic balance of the internal state as evident from the hemodynamic response signal.

The prefrontal cortex was the sole part of the brain that was investigated in this study because of fewer number of fNIRS channels present. To broaden the field of inquiry, future studies can enhance their exploration of various brain regions by using additional channels. In addition, the integration of hybrid EEG-fNIRS neuroimaging techniques offers a potential approach to capture brain signals, allowing for a comprehensive study of the entire brain and validation of the current study’s hypotheses [74,75]. It is important to recognize a key drawback of fNIRS, which is the heterogeneity in the hemodynamic response signal between different individuals and within the same individual [76]. This limitation was overcome by combining data from multiple participants, allowing for a comprehensive assessment of the brain response patterns. To improve spatial resolution and increase the accuracy of activation maps, future research could investigate the positioning of optodes at high densities or in bundles, incorporating short separation channels [77].

One more factor that might have affected the fNIRS data is fatigue due to the fNIRS equipment. The experimental design included measures to minimize participant fatigue. However, the setup and wearing of the fNIRS cap could have contributed to participant discomfort or fatigue during the session. Fatigue may affect participants’ cognitive performance and stress levels, potentially influencing task outcomes and hemodynamic responses. While efforts were made to ensure comfort, such as using lightweight fNIRS equipment, it is possible that residual fatigue might have influenced the results. Future studies could employ additional strategies, such as shorter task durations or the use of more ergonomic equipment, to further mitigate the impact of fatigue on the brain signals.

Subsequent studies could investigate the neuroplasticity occurring within a few hours or days after tACS therapy in order to ascertain its enduring impact. Another limitation pertains to the sample size of the study, which, while similar to previous studies, suggests the potential for expansion to a larger group. Moreover, this study did not examine gender variability. One potential avenue is to conduct separate research on male and female individuals to provide comparison analysis. Furthermore, future research could focus on implementing alternative forms of electrical or magnetic stimulation exclusively on specific brain regions to assess their distinct impacts on different brain regions [78,79]. Future research should explore the long-term benefits and broader applications of tACS to further establish its clinical significance.

## 5. Conclusion

In this study, transcranial alternating current stimulation (tACS) has been utilized to improve brain function and to lessen psychological stress. Except for a few cases of tingling and fatigue, the study shows that tACS is a safe and practical procedure with little side effects. While the sham group reported feeling bored and exhausted, those getting tACS reported feeling more focused. The hemodynamic response data revealed an increase in brain activation following tACS, unlike the sham stimulation, which resulted in diminished brain activation. This was further supported by t-maps showing enhanced activation in the dorsolateral prefrontal cortex post-tACS. In contrary, the study also observed a substantial decrease in functional connectivity following tACS. Overall, these findings underscore the potential of tACS as an effective non-invasive modulation technique for improving brain function and managing mental stress.
